# Supplementary material for: Dancing for Parkinson’s Disease Online: Clinical Trial Process Evaluation
Source: Healthcare (Basel). 2023 Feb 17;11(4):604. doi: 10.3390/healthcare11040604 (PMC9957486; doi:10.3390/healthcare11040604)
Supplement: Supplementary file 1 [file healthcare-11-00604-s001.zip › healthcare-2186721-supplementary.pdf]

ParkinDANCE ONLINE

GUIDELINES FOR NEUROLOGICAL ASSESSORS

INTRODUCTION AND BACKGROUND

Participants in this study are being assessed at two time points, approximately six weeks apart, using both questionnaires and objective outcome measures. All assessments will be completed via online video sessions involving the Assessor and individual participants

A video-conferencing platform will be used. Familiarity with using this platform is essential for the Assessors prior to undertaking the participant assessments. The Assessors will complete an Orientation session with participants to familiarise them with the video-conferencing program and the assessment procedures.

Each assessment will take about one hour to complete.

All participants have Parkinson's Disease with comparatively mild symptoms (Hoehn and Yahr scores 0-2.5). They can all walk independently but may have impaired balance.

PREPARATION FOR THE ONLINE INTERVIEWS

- **Set up of your interview space**.
    - Check your image in the camera before you start the interview. Are you clearly visible? Make sure that there is a light source on your face, and you are not just a dark shape in front of a window. Ensure that the camera on the computer is at about eye height, not looking up your nostrils! You may need to raise your laptop on a box or books.
    - Check that your background is professional-looking and that family members will not be entering the space during the assessment. These are issues of confidentiality and privacy for the participant.
    - Ensure you have your workspace organised so you can record the participants' answers, while still maintaining your image in the camera.
- **Participants' telephone number**. Before commencing the video-conference session, the assessor should make sure that they have a phone number for the participant on hand, in case there are any connection problems in the session.
- **Enhance connectivity.** Minimise the number of open programs running on your computer and check that no one else using your modem is streaming data at that time, to optimise the audio and visual quality of the video-conference session.
- **Check audio-visual quality**. At the start of the session, introduce yourself and ask about the audio and visual quality of the session from the participant's perspective. Can they see and hear you clearly? Check for time delay by counting one, two, three out loud as you demonstrate with your fingers, to make sure the sound and vision that they receive is synchronised.

COPYRIGHT

- **Communication style**. You may need to adapt your verbal delivery by speaking a little slower and louder than you normally would, and increasing your facial expressions and tonal variation to enhance your communication using video.

INITIAL SESSION: ORIENTATION

The assessor will conduct a pre-assessment orientation session online with each participant. The goals of this session are to familiarise the participant with the technology and also work out the logistics of the performance measures (single leg stance and six metre walk test). A further purpose of this orientation session is to help alleviate any anxiety the participant may have about engaging in this unfamiliar form of communication.

Technology familiarisation:

- This may be the first time the participant has done a Zoom meeting. The Assessor is advised to ring the participant to organise a time for this session, send through the Zoom invitation via email, then give the participant a reminder phone call 10 minutes before the meeting is due to start. It may be necessary to also talk to the participant on the phone to trouble shoot during this initial meeting as there may be difficulties with processes such as opening the invitation, joining with video, unmuting the microphone or adjusting the volume of the speaker on the computer/iPad.
- Orientate the participant to various features of the zoom meeting window – such as the speaker/gallery view and enter/exit full screen.

Single leg stance timed test:

- The whole of the participant's body needs to be visible to the Assessor for this test, including the feet. The participant may need to step back from the computer a few metres and adjust the angle of the screen. The aim in this orientation session is for the participant to identify the ideal position to stand and the ideal screen setup angle, so that when doing this test in the formal assessment, the participant can just concentrate on the balance task, and not be distracted by the set up details.

Six metre walk test:

- This test involves walking along a six metre walkway while being timed. This may be challenging in the home setting. Ideally there would be a six metre empty runway behind the computer that the participant can walk down and be timed doing so. In reality, the only six metre runway (e.g. a hallway) may be out of view of the computer. If this is the case, a family member would be needed to record the start and finish of the timed walk. If this is not possible, the test may not be able to be done. A self-timed test is not preferable, and may not be included in data analysis.
- Measuring the six metres must be done accurately. For the purpose of the orientation session, it can be paced out as 6 long strides – to try to find a suitable place in the home. Before the formal assessment, the participant needs to measure this out with a tape measure and mark the start and finish with tape on the floor. Ideally there should be extra

COPYRIGHT

space at the start and finish of the walk for acceleration and deceleration. For people with Parkinson's Disease, extra space at the end is particularly important, so they don't slow down before they reach the end.

- Check if the participant has a tape measure suitable for measuring six metres. If they don't have one, the university can provide them with one prior to the assessment. For this reason, the orientation session should be scheduled as least a few days before the assessment date, to allow time for delivery of one.
- Record all the details of where the test was done and who recorded it, to make sure the same conditions are replicated on re-testing.

TESTING PROCEDURE

- Complete every page of the Assessment Manual using a black pen, including the date and code at the top of every page.
- To ensure that the participant environments are safe, participants will have completed a self-assessment to identify potential hazards in the environment e.g. space, flooring, obstacles, rugs or mats, stable chair for seated or assisted exercise and shoe type. During the assessment, the Assessor should check the environment to ensure it is indeed safe and the participant has completed the hazard identification survey accurately.
- Ensure that both audio and visual connection is good for both the assessor and the participant.
- A digital stop watch is required for timed tests (a stop watch on a phone is acceptable).
- A carer /partner may be present, but communication should be directed to the participant, and the carer/partner just included for clarification as required.
- At all times, participant safety is of paramount importance, and the assessment should be stopped at any point the assessor is concerned about participant safety. Write "Not completed" on the Assessment form, and the reason that there is no score for that test should be recorded on the Assessment form.
- Online communication via video can be tiring as it requires sustained concentration. The assessor should offer the participant a short break after 30 minutes. One or two minutes to relax / stand up / get a drink is suggested.
- Consumer input about their experience will be conducted with each participant via telephone or open-ended survey at the end of the study.

OUTCOME MEASURES

- Single leg stance test (timed test)
- Six metre walk test (timed test)
- Disability using the MDS-UPDRS (questionnaire)
- Quality of life using the PDQ39 (questionnaire)

Specific instructions relating to these outcome measures are including in the Assessment Manual. Record the results of each test directly onto the Assessment Manual.

Please note that there are two items on the UPDRS that cannot be tested online (3.3 Rigidity and 3.12 Postural Stability). These items have been identified as 'Not for testing' in the Assessment manual.

COPYRIGHT

CONTEXT

A range of contextual factors shaped the design, implementation, mechanisms, evaluation and outcomes of ParkinDANCE Online© and have been measured in the project. These included: (i) a review of global literature on dancing for Parkinson's disease and consideration of the broader literature on exercise, physical activity, and physiotherapy, to guide choices about dance genres, music selections, class content, dosage and digital delivery (ii) stakeholder engagement with the Parkinson's community, industry partners, implementation scientists, dance teachers and health professionals (iii) a commitment to co-design of ParkinDANCE with individuals living with Parkinson's disease and the significant others in their lives, as well as the local Parkinson's disease organisation (iv) consideration that the ParkinDANCE Online© research project was introduced during the COVID-19 pandemic, necessitating a rapid pivot from group community classes to one-to-one online classes. Research processes also needed to be adapted to online delivery and monitoring. Staff required rapid training and support to ensure safe, effective and competent implementation of all elements of the project online.

DESCRIPTION & ASSUMPTIONS

ParkinDANCE Online©

IMPLEMENTATION

Trial processes:
- How delivery was achieved
- Training
- Resources

What was delivered:
- Dose
- Fidelity
- Adaptations
- By and to whom

MECHANISMS OF IMPACT

- Participant and stakeholder co-design and feedback on the program and delivery
- Mediators such as access, training and co-production
- Unanticipated mechanisms, facilitators, barriers and consequences

OUTCOMES

LA TROBE UNIVERSITY

ARCH Academic and Research Collaborative in Health

Fight Parkinson's Together we can

Supplementary File s2 Morris et al (2023) Program Logic Model

Based on Fig 1. Moore et al. BMJ 2015;350;h1258 doi: 10.1136/bmj.h1258

ParkinDANCE ONLINE

PARTICIPANT GUIDE

Contents

1 – Introduction 1
WELCOME 1
MEET THE TEAM 1
2 – Setting up for a class 2
EQUIPMENT YOU WILL NEED 2
ENVIRONMENT FOR DANCE SPACE 2
CLOTHING & FOOTWEAR 3
SETTING UP ZOOM 3
3 – Class Format 4
WHAT TO EXPECT FROM A SESSION 4
ACTIVE DANCE PHASE 4
COOL DOWN 4
ASSESSMENT SESSIONS 4
4 – Safety Considerations 5
PROCEDURE TO FOLLOW IF A FALL OCCURS 5
FEELING DIZZY OR FAINT 5
SUDDEN DETERIORATION IN SYMPTOMS AND POSSIBLE CAUSES 6
SIDE EFFECTS AND BODY REACTIONS TO EXPECT 6
"ON AND OFF" PERIODS 6
5 – Contact details 7

COPYRIGHT

ParkinDANCE ONLINE

1 – Introduction

WELCOME

Welcome to the Dance Exercises for Parkinson's Rehabilitation (Online): How Much is Enough study! [redacted] you are eligible to take part. We would like to welcome you to the study and thank you for volunteering your time.

You will have a short orientation meeting with the Project Manager before starting the study. This will be to introduce the study and explain what to expect.

MEET THE TEAM

[redacted]

[redacted]

[redacted]

Our Team of Qualified Dance Instructors

COPYRIGHT

ParkinDANCE ONLINE

2 – Setting up for a class

EQUIPMENT YOU WILL NEED

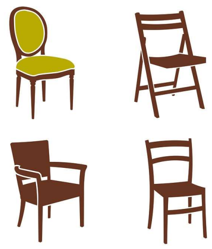

1. A stable chair – a kitchen or dining room chair is best, with no castors or wheels. It should be low backed so that your hands can rest comfortably on the top of the chair back.
2. A computer, laptop or tablet device with internet access and webcam facility. We recommend that you test your internet connection and speed before your first session to ensure it can support zoom. You will need to be hands free to do the dance moves, so we do not recommend using a smartphone.
3. Zoom installed on your device. You will need to be familiar with the speaker and volume settings or have someone who can help with this.
4. A bottle of water for each class.
5. Your mobile or telephone phone within reach.

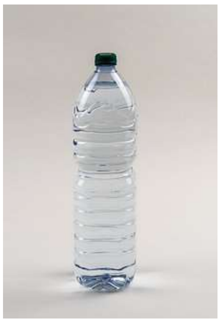

ENVIRONMENT FOR DANCE SPACE

- At least a 2m x 2m clear indoor space, in front of your device. Your dancing area needs to be clear of furniture, obstacles and clutter including cables, cords, loose mats and carpets.
- The flooring area must not be wet or highly polished. Check for cracks, nails or surface irregularities that you could step on.
- Your device needs to be positioned on a table at a height that you can clearly see the screen when you are standing up and sitting down. We recommend trialling this before your first session as getting the positioning right may take a few adjustments.
- Ensure the area is well lit and is not too hot or cold. Remember, you may warm up during the class.
- Limit distractions during the dance session. This may include background noise such as TVs or radios, or devices such as telephone calls and answering machines.
- Limit interruptions, such as other members of the household or young children coming into the room.
- Secure any pets in another part of the home.

COPYRIGHT

ParkinDANCE ONLINE

CLOTHING & FOOTWEAR

Wear	Avoid wearing
• Firm fitting shoes with fasteners or laces<br>• Shoes with non-slip soles<br>• Loose-fitting, comfortable clothing<br>• Active wear or tracksuit pants	• Open backed shoes<br>• Slide in shoes or slippers<br>• Bare feet<br>• Stockings or tights with no shoes<br>• Tight or restrictive clothing<br>• Long hemlines, such a skirts or dressing gowns

SETTING UP THE VIDEO-CONFERENCE

- Ensure the application is installed on your device prior to your first session. We will use the Zoom platform.
- Click on the link in the email for that class session. Zoom will load automatically, follow the prompts to access the session.
- Turn your computer volume up and ensure your webcam and speakers are on.
- If you are experiencing problems and the class has already started please contact the Project Manager

COPYRIGHT

ParkinDANCE ONLINE

3 – Class Format

WHAT TO EXPECT FROM A SESSION

One-to-one classes will last for 1 hour. There will be 2 classes a week for 4 weeks at times that are negotiated for your convenience and peak medication dosage. From time to time a qualified physiotherapist will join the session.

WARM UP

5-minute warm-up which will be directed by the dance instructor.

ACTIVE DANCE PHASE

30 minutes active dance phase in sitting, standing and moving which will be directed by the dance instructor. There will be a range of accompanying music that cues the dance movements. You will be assigned one instructor for the entire 4 weeks. The sessions will cover a variety of dance types including: Tango, Tap, Ballroom, Salsa and Creative.

Rests and drink breaks will be provided.

COOL DOWN

5-minute cool-down which will be directed by the dance instructor.

ASSESSMENT SESSIONS

As well as the dancing classes there will also be an online videoconference assessment before and after the classes commence. There will be a brief introductory videoconference meeting with the assessor before the first assessment (approximately 15 mins). This will be followed by an assessment session of approximately 1 hour that is scheduled at a convenient time for you. The assessor is a qualified physiotherapist who does not know the details of the dance sessions.

COPYRIGHT

ParkinDANCE ONLINE

4 – Safety Considerations

PROCEDURE TO FOLLOW IF A FALL OCCURS

There is a small risk of falls during the online classes. We aim to prevent this by teaching you, the dance teachers and assistants how to balance and prevent falls. Many of the dance and exercise activities are sitting in a chair or standing holding on to the back of a chair, or balancing using walls for support.

If a fall does occur, please follow these steps:

1. Try to remain calm and do not attempt to get up straightaway.
2. Call for help from anyone present in your household, we will have a next of kin contact that we can call. We can also call an ambulance if needed.

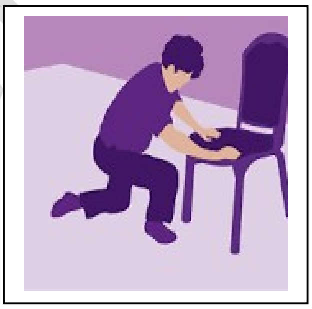

3. Perform a self-assessment for injuries by being aware of any pain, looking and feeling for any injuries.
4. If you can, slowly sit up and remain in this position for a few minutes.
5. If you can, turn on to your hands and knees and use the seat of your chair to slowly stand up.
6. Sit onto your chair and the research team will contact you.

FEELING DIZZY OR FAINT

If you feel dizzy or faint during a class, follow these steps:

1. Sit down
2. Drink some water
3. Have a snack if you need to
4. Take slow, deep breaths
5. A member of the research team will contact you

COPYRIGHT

ParkinDANCE ONLINE

SUDDEN DETERIORATION IN SYMPTOMS AND POSSIBLE CAUSES

If you have a sudden change in your symptoms, this may affect your safety in the class. You should consult your GP. Reasons for this may include:

- Illness or infection
- Stress, anxiety, depression or mood changes
- Constipation
- Dehydration
- Pain
- Changes to medications regime or use of contraindicated drugs

SIDE EFFECTS AND BODY REACTIONS TO EXPECT

- Increased temperature, breathing rate and heart rate are all normal reactions to exercise. We will monitor these changes during the class.
- Mild muscle soreness after exercise can normally be experienced for up to 48 hours We shall design the dance exercises to be suitable for your abilities.
- Our team will always be contactable to answer any questions about side-effects you may feel during or after a class.

"ON AND OFF" PERIODS

This relates to the reaction of your symptoms to your medication. You may have "ON" periods where your movements are optimised by your medications. "OFF" periods describe the deterioration of symptoms as your medications wear off. We recommend attending a class during an "ON" period in your medication regime. Usually the peak dosage is about one hour after taking your medication.

COPYRIGHT

ParkinDANCE ONLINE

5 – Contact details

[redacted]

THANK YOU AND ENJOY!

COPYRIGHT

ParkinDANCE ONLINE

MANUAL FOR DANCE INSTRUCTORS

Parkinson's Disease affects a person's movement; they may experience stiffness, slowness, impaired coordination and shaking. There is no cure, but some people may be using a drug Levadopa (L Dopa) that gives temporary relief. For more information see: www.parkinsonsvic.org.au   All participants in this study have Parkinson's Disease with relatively mild symptoms. They can all walk independently, but may have impaired balance.

Dance classes have been shown to benefit people with Parkinson's Disease, to help with their balance and mobility. This study is exploring the use of live online dance classes. In this first phase of the study, the instructor will be one to one with each participant for four classes.

The video-conference platform will be used. Familiarity with using this platform is essential for the Instructors prior to teaching the classes. If the Instructor is not familiar with using it, practise with a colleague first is highly recommended.

Each class will take about one hour – greetings and health check; warm up; active component; cool down; and a short online feedback survey will be presented to participants and to instructors at end of zoom sessions.

AIMS OF THE DANCE CLASSES:

- Improve balance:
    - Improve ability to stand steadily on various bases of support, i.e. feet placed in different arrangements – together, apart, on one leg when stepping etc.
    - Improve ability to move the body and shift weight in every direction – forward/backward; side to side; diagonals; rotation.
    - Improve ability to take a step in any direction (to protect oneself from falling).
    - Reduce rigidity of trunk and upper limbs to allow postural readjustments.
- Increase confidence, and reduce anxiety, about moving through space
- Improve body awareness and spatial awareness (both which can become diminished in PD)
- Improve cardiorespiratory endurance (as participants may be quite unfit)
- Improve strength of lower limbs
- It can be useful to think about which of these goals are being addressed in the various dance activities.

COPYRIGHT

ParkinDANCE ONLINE

PREPARATION FOR TEACHING THE ONLINE CLASS

Set up of your interview space.

Check your image in the camera before you start the session. Are you clearly visible? Make sure that there is a light source on your face, and you are not just a dark shape in front of a window. Ensure for your initial greeting and check-in that the camera on the computer is at about eye height, not looking up your nostrils! You may need to raise your laptop on a box or books. **Note: this may be slightly different to how the participant is required to appear** (see Figure 1 and 2).

[Grab your reader's attention with a great quote from the document or use this space to emphasize a key point. To place this text box anywhere on the page, just drag it.]

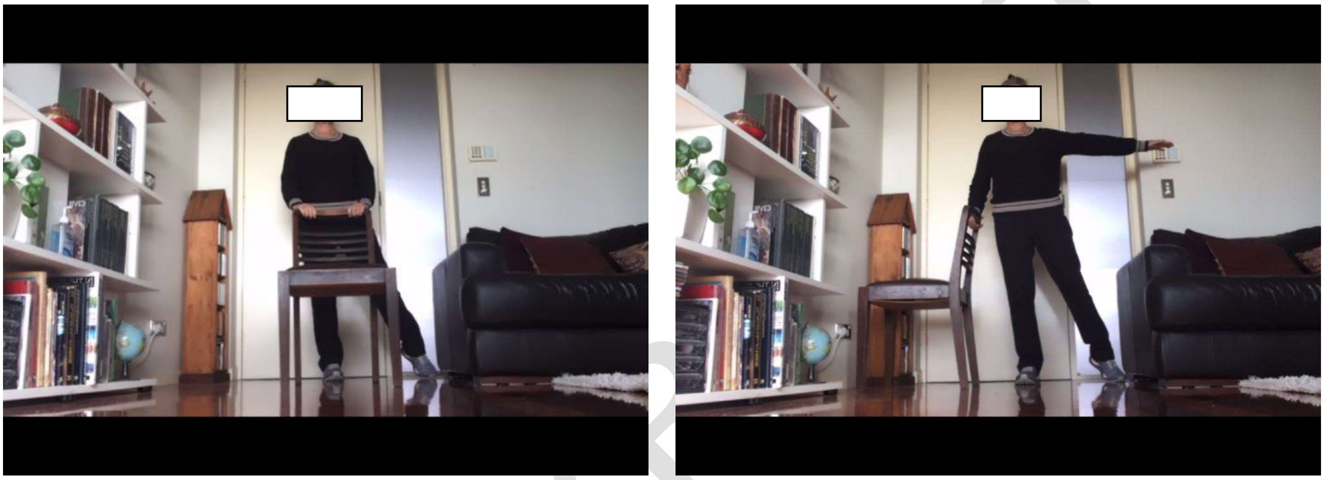

Figure 1. Example of set up required for participant – sturdy chair (positioned either in front or to the side), participant visible on screen from head to feet. In this set up the iPad was on the floor 3 metres away.

Once you move to the more active part of the class, ensure that your whole body is visible to the participant (this may require adjustment of your screen angle).

Ensure that your microphone can pick up your voice well when you are demonstrating movements, especially if you also have music playing. You may need a lapel microphone or enhance audio via the video-conference software.

(Note: To optimise your audio from computer you can share your audio as part of the share screen function: https://youtu.be/sYWZqXnqrSY)

Check that your background is professional-looking and that family members will not be entering the space during the assessment. These are issues of confidentiality and privacy for the participant.

ParkinDANCE ONLINE

Participants' telephone number. Before commencing the video-conference session, the Dance Instructor should make sure that they have a phone number for the participant on hand, in case there are any connection problems in the session.

Enhance internet connectivity. Minimise the number of open programs running on your computer and check that no one else is using your modem to stream data at that time, to optimise the audio and visual quality of the videoconference session.

Video-conference invitation. Once the class days and times have been agreed between the Dance Instructor and the Participant the Dance Instructor will send recurring online invitations to the participant and the Project Manager. It is important to check that these invitations are accepted and in the calendar.

Check audio-visual quality. At the start of the session, introduce yourself and ask about the audio and visual quality of the session from the participant's perspective. **Note: you can optimise audio quality by sharing your audio zoom (see here for details on how to do this: https://youtu.be/sYWZqXnqrSY).** Can they see and hear you clearly? Check for time delay by counting one, two, three out loud as you demonstrate with your fingers, to make sure the sound and vision that they receive is synchronised.

Communication style. You may need to adapt your verbal delivery by speaking a little slower and louder than you normally would, and increasing your facial expressions and tonal variation to enhance your communication using video.

COPYRIGHT

ParkinDANCE ONLINE

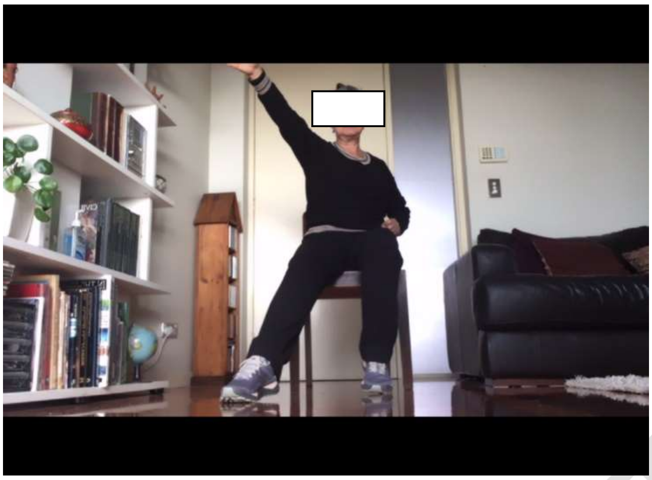

Figure 2. Example of set up required for participant – sturdy chair (positioned either in front or to the side), participant visible on screen from head to feet. In this set up the iPad was on the floor 3 metres away.

CONDUCTING THE CLASS

Greetings. Introduce yourself and explain the format of the class, so the participant knows what to expect. They may be feeling anxious, which will impact on their ability to move safely, so reassure them that you will be watching them and adjusting the class to suit their abilities. Remind them that the class will be fun and you want them to enjoy it. Also invite their feedback at any time, particularly if they are feeling unsafe or unwell.

Health check in. People with Parkinson's disease can vary a lot from day to day, even hour to hour depending on their medication. Check when they last had their medication, to make sure it's not almost worn off. It may be best to time the dance class when their medication is having its best effects. Ask how they are feeling today. Also enquire about their recent health - have they had any falls? Have they been unwell? If they report feeling unwell / dizzy/ in pain/ injured from a fall, do not proceed with the dance class. Let them know that you will check with the Study supervisor and get back to them.

Environment. Participants will have completed an environmental safety check. During the class, make sure that the area is safe – enough space, suitable chair, no mats or cables to trip on, suitable footwear, no pets around.

Continuously observe the participant. Safety is of utmost importance, as these participants are at risk of falls. Sometimes when dancing, it is easy to get involved in concentrating on your own movements, however in this situation you must always be observing the participants keenly,

COPYRIGHT

especially when they are on their feet. If they look at all unsteady, encourage them to hold onto the back of the chair and/or move more slowly and/or change to a different, less challenging movement task.

Adverse events. If a fall occurs or the person injures themselves, this must be reported to the [redacted]. If the injury is serious (for example if they can't get up from the floor), call an ambulance [redacted] and also notify the person's emergency contact. Any adverse events, even minor ones such as muscle soreness following the previous class, need to be recorded on the participants' Attendance sheet.

If a participant does fall during the class, instruct the person to take their time and not rush to try to get up. They should check themselves for any injury, and if they are OK, they should get onto their hands and knees then use a chair to help themselves back onto their feet. Do not continue the class. Advise them to go and rest, and someone from the research team will check on them later in the day.

COMPLETION OF THE CLASS

Immediate feedback. Ask the participant how they thought the class went and if they have any immediate feedback. Record this information on the participant's Attendance sheet. The participant (and instructor) will also receive a short anonymous online survey at the end of each zoom session, please remind the participant of this survey *and that it is anonymous* before saying good bye for each session.

Final feedback. Remind the participants that they will be contacted for feedback by the study organisers at the end of the series of classes, and encourage them to participate in that as well as the short online surveys at the end of each class.

Next class. Confirm the date and time of the next class.

Reflections. After finishing the v ideo-conference meeting, complete the online survey you will receive after the end of the session. Also use the form at the end of this manual to record impressions and any other information which may be useful, such as things that did go well, or did not go so well, and how you might do something different next time. This information will be useful for the interview you will have with the research team at the completion of the classes

COPYRIGHT

ParkinDANCE ONLINE

COPYRIGHT

ParkinDANCE ONLINE

SAMPLE CLASS TEMPLATES

The tables below include samples from four different dance approaches, to demonstrate examples of dance activities undertaken in each phase (warm up, active component and cool down) of the classes.

Warm up and cool down phases will be approximately 5 minutes each.

The active component will be 30 minutes (break down: approximately 8 minutes, 3 minutes break, 8 minutes, 3 minutes break, 8 minutes)

NOTE: avoid extreme or end of range joint movement; avoid neck rotations, forward and backward bending. Ease slowly into each movement.

WARM UP (approximately 5 minutes)

VERSION	AIMS	DANCE	POSITION	MUSIC
Creative dance	Focus and breath, to commence feeling relaxed & leaving distractions behind	Gentle, expansive movements of the upper limbs and mobilising movements of the lower limbs.	Sitting	Piano sonata No14 in c-sharp-Minor, Ludwig Beethoven
Tango inspired	Awakening and activating body	Gentle torso twist, side stretch, stepping on spot, knee raise, knee raise & kick, circle foot front, circle foot back, double time kicks	Sitting	Recuerdo, Richardo Tanturi
Ballet based	Relaxation, breathing, self-massage, slight rotation & coordination	Eyes, head, hands, arms, torso	Sitting	Could it be magic, David Plumpton*<br><br>*David Plumpton's tracks are created for dance classes.
Latin / Ballroom	Prepare body, breathing, relaxation, large amplitude, coordination	Posture check, heel lifts, steps, arms, body, spinal flexibility	Standing	Fantasy – Del

COPYRIGHT

ParkinDANCE ONLINE

ACTIVE COMPONENT OF CLASSES (approximately 30 minutes)

VERSION	AIMS	DANCE	POSITION	MUSIC
Creative dance	Lower body strength, balance, co-ordination, stability, cardiovascular conditioning	Marching steps.<br><br>Forward step & back to centre.<br>Step back with other leg & back to centre.<br><br>Cha Cha Cha step<br><br>Glide and slide	Standing next to chair	Tea for two, Cha Cha Cha
Tango inspired	Strengthening, aligning & balance	Heel raises, heel raise jiggle knees, alternate legs. Mini squats.<br>Step to side, then balance on side step, continuing stepping side to side but tapping with free leg	Standing	Isla de Capri, Osvaldo Fresedo
Ballet based	Co-ordination, balance, strength, posture	Demi plie; Port de Bras; stretches	Standing – with acting ballet barre (stable chair/bench top/ kitchen table)	My heart will go on, David Plumpton
Latin/ballroom	Co-ordination, gait, combination upper & lower body movement, balance	Basic rumba	Standing	Afro-cuban background music

COPYRIGHT

ParkinDANCE ONLINE

COOL DOWN (approximately 5 minutes)

VERSION	AIMS	DANCE	POSITION	MUSIC
Creative dance	Focus on breath to return to natural rhythm, stretches, relaxation	Arms float up and down (inhale on the up/exhale on the down), spinal twisting, gentle movements of lower limbs (foot circles, knee straightening)	Sitting	Ain't no sunshine, Eva Cassidy
Tango inspired	Lower body, volume movements	Slow movements of legs and weight shifts	Standing	El Gitarrero, Argentinian music
Ballet based	Breathing, rotation, stretch, relaxation, clarity	Head, arms, torso, spine gentle stretches.	Sitting	Frozen, David Plumpton
Latin / ballroom	Coordination and loosening of body	Lower limb movements. Upper body circular motion, soften spine curl. Gently raise arms and bring back to sides	Sitting	Todo es Amor, Orquesta Romantica Milonguera

COPYRIGHT

ParkinDANCE ONLINE

PARTICPANT ATTENDANCE AND DANCE INSTRUCTOR FEEDBACK

PARTICIPANT ID NUMBER:

Class date	Attendance	Comments in addition to automated online survey

**Adverse Event/s** *(include date, what happened, action taken and outcome)*	
**Reflections** *(immediately after the four-week intervention)*	

COPYRIGHT

ParkinDANCE ONLINE

Instructor name (PRINT)	
Instructor signature	Date

PARTICPANT ATTENDANCE AND DANCE INSTRUCTOR FEEDBACK

**PARTICIPANT ID NUMBER:**		
**Class date**	**Attendance**	**Comments in addition to automated online survey**

**Adverse Event/s** *(include date, what happened, action taken and outcome)*	
**Reflections**	

COPYRIGHT

ParkinDANCE ONLINE

(immediately after the four-week intervention)		
Instructor name (PRINT)		
Instructor signature		Date

COPYRIGHT

Fight Parkinson's Together we can

LA TROBE UNIVERSITY

ParkinDANCE ONLINE

Video-conference guide for Dance Exercises

We recommend using a computer, laptop or tablet for your assessment and dance sessions, not a smart phone. This is so that you can place it at the correct height, have 2m x 2m space in front of it and still be hands free. Please also choose a device that has a big enough screen to ensure you can see your dance instructor clearly.

Installing video-conferencing platform if you do not have it installed on your device

Zoom is a free program that enables video calls between people. It needs to be downloaded to your device, before it can be used for video calls. It can be downloaded to Windows and Mac devices. **If you usually require support accessing IT, please make sure someone is with you that can help.**

1. Go to https://zoom.us/download
2. Click on the 'Zoom Client for Meetings' download button.
3. The Zoom installer will start to load, click on this to run the download. Follow the instructions to download.

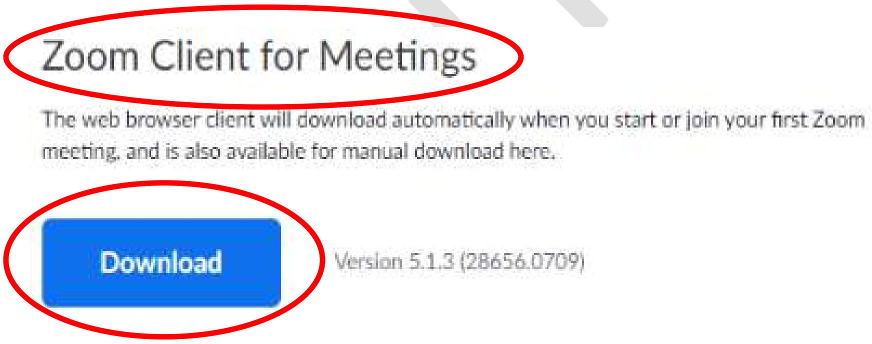

4. Zoom will now be installed on your device
5. You may be prompted to sign up and activate your account. Do this by using your email address and choosing a password for your zoom account. You will receive an email from zoom. Once you receive this email, click Activate your zoom account.

PLEASE NOTE ZOOM IS A FREE APP AND YOU SHOULD NEVER NEED TO PAY TO USE IT

Accessing your assessment or dance session

You will receive an email invitation with a link to your zoom session from the Project Manager [redacted] Please make sure it is the correct day and time.

Please click on the link and follow the instructions for the session to load.

You may need to enter a passcode to enter the session, this will be provided in the email invitation.

Remember to join WITH computer audio

ParkinDANCE ONLINE

Once you have accessed your session on Zoom – testing video and audio

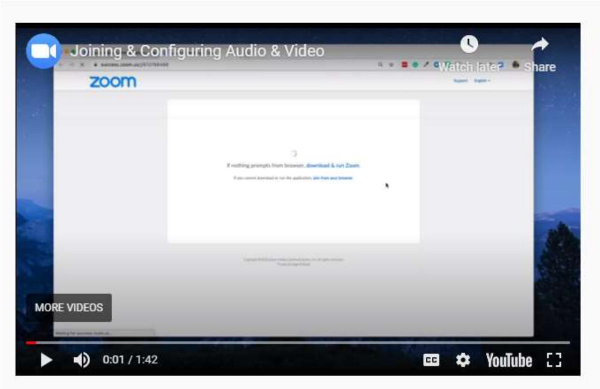

Please watch this YouTube tutorial by the zoom help centre for instructions about how to test and adjust your video and audio; below link can be cut and pasted into your browser:

https://www.youtube.com/watch?v=-s76QHshQnY

Enhancing Sound

There are two ways to enhance the sound for classes:

1. Purchase speakers (low cost at places like Officeworks) and plug into computer or laptop
2. Instructors can also optimise audio via zoom: https://youtu.be/sYWZqXnqrSY

Updating your version of zoom

To ensure you are able to access the assessment and dance classes, you must be using the latest version of zoom. To do this:

1. Sign in to zoom desktop app
2. Click on your profile picture
3. Click Check for updates
4. If there is a newer version, zoom will download and install it

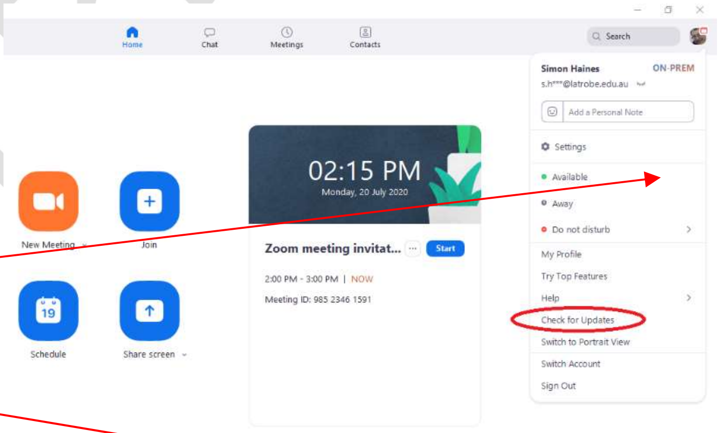

COPYRIGHT

Zoom support

If you are experiencing any technical problems, you can contact the Project Manager [redacted] who can provide 1:1 support.

ParkinDANCE ONLINE

COPYRIGHT
